# Gadd45α: A Novel Diabetes-Associated Gene Potentially Linking Diabetic Cardiomyopathy and Baroreflex Dysfunction

**DOI:** 10.1371/journal.pone.0049077

**Published:** 2012-12-05

**Authors:** Ning Wang, Chao Yang, Fang Xie, Lihua Sun, Xiaolin Su, Ying Wang, Ran Wei, Rong Zhang, Xia Li, Baofeng Yang, Jing Ai

**Affiliations:** 1 Department of Pharmacology, Harbin Medical University (the State-Province Key Laboratory of Biomedicine-Pharmaceutics of China), Harbin, People's Republic of China; 2 Key Laboratory of Cardiovascular Research, Ministry of Education of China, Harbin, People's Republic of China; 3 College of Bioinformatics Science and Technology, Harbin Medical University, Harbin, People's Republic of China; University of Otago, New Zealand

## Abstract

Both diabetic cardiomyopathy (DCM) and baroreflex dysfunction independently contribute to sudden cardiac death (SCD), however the inherent connections between them under diabetic state remains unclear. As microRNAs (miRNAs) have been reported to participate in various physiological and pathological processes, we presume they may also be involved in DCM and DM-induced impairment of baroreflex sensitivity. Two sets of gene expression profiles data from streptozotocin (STZ)-induced diabetic heart and diabetic dorsal root ganglia (DDRG) were retrieved from GEO and ArrayExpress. Co-differentially-expressed genes in diabetic heart and DDRG were identified by *t* test and intersection analysis. Human Protein Reference Database (HPRD) was applied to find direct interacting proteins of Gadd45α. Differentially-expressed miRNAs in left ventricle from 4-week STZ-induced diabetic rats were screened by miRNA microarray. Expression of miR-499 and its regulating effect on Gadd45α were then verified by quantitative real-time PCR (qRT-PCR), western blot, computational predication, and dual-luciferase reporter analysis. Four co-differentially-expressed genes in DCM and DDRG were identified. Among these genes, Gadd45α has 16 direct interacting proteins and 11 of them are documentedly associated with DM. Accompanied with significantly increased miR-499 expression, Gadd45α expression was increased at mRNA level but decreased at protein level in both diabetic heart and nucleus ambiguous. Furthermore, miR-499 was confirmed negatively regulating Gadd45α by targeting its 3′UTR. Collectively, reduced Gadd45α protein expression by forced miR-499 expression indicated it's a diabetes-associated gene which might potentially be involved in both DCM and DM-induced baroreflex dysfunction.

## Introduction

Diabetes mellitus (DM) is an ever-growing problem nowadays, and the number of diabetic adults worldwide is estimated to be 300 million in the year 2025 [1]. Sudden cardiac death (SCD) is the most serious outcome of DM, and clinical data suggested that DM carried a hazard ratio of 3: 23 for SCD [2]. Among the complications of DM, diabetic cardiomyopathy (DCM) and diabetic cardiac autonomic neuropathy (CAN) were reported to be closely associated with SCD in DM [3], [4], moreover positive correlation has been established between DCM and diabetic CAN [5], [6]. Although substantial efforts have been devoted to revealing the participation of DCM or DM-induced baroreflex dysfunction in SCD, the common inducer contributing to both DCM and impaired baroreflex sensitivity has not been well studied yet. Undoubtedly, investigating the co-differentially-expressed genes in diabetic heart and baroreflex circuitry would be an optimized approach to find the linker between DCM and diabetic baroreflex dysfunction.

MicroRNAs (miRNAs) are short noncoding RNA molecules playing critical roles in posttranscriptional regulation by inhibiting messenger RNA translation or specially cleaving them [7]. Numerous studies have revealed obvious associations between altered miRNA expression and some diabetic complications [8]. Furthermore, many miRNAs have been reported to play a role in diabetic heart, such as miR-1 [9], miR-133a [10], and miR-320 [11]. Nevertheless, whether miRNAs could regulate the “linker” genes between DCM and DM-induced baroreflex dysfunction and hence contribute to SCD is still undetermined.

The present study suggests that co-differentially-expressed miR-target pair, miR-499::Gadd45α, might be involved in the tissue-tissue communication between DCM and DM-induced baroreflex dysfunction by an innovative incorporation of bioinformatics, miRNAs microarray analysis and biological experiments, and therefore provides a potential preventive strategy for SCD in DM.

## Methods

### Ethics Statement

The study was performed in strict accordance with the Guide for the *Care and Use of Laboratory Animals of the National Institutes of Health*. The protocol was approved by the committee on the Ethics of Harbin Medical University (No. HMUIRB-2008-06).

### Gene microarray data analysis and hierarchial clustering

In this study, we analyzed two sets of gene expression profiles. One was downloaded from Gene Expression Omnibus (GEO) (http://www.ncbi.nlm.nih.gov/geo/) and derived from a study on DCM with 7 samples for control (Ctl) and 7 samples for DCM rat hearts (accession number GSE5606) [12]. The other microarray dataset was downloaded from ArrayExpress database (http://www.ebi.ac.uk/arrayexpress/) (accession ID: E-MEXP-515) [13] which detected the gene expression of 17 samples: 3, 3, and 3 samples obtained from the dorsal root ganglia (DRG) of 1, 4, and 8-week streptozotocin (STZ)-induced diabetic rats, and 2, 3, and 3 samples from age-matched Ctl rats. We normalized the microarray data using Arraytools (http://linus.nci.nih.gov/BRB-ArrayTools.html). Comparison of gene expression levels between experimental group (1, 4, 8-week DDRG and 16-week DCM; DDRG: diabetic DRG) and the age-matched Ctl group by separate *t* tests. After performing significance analysis of microarray, those showing a significantly different expression (*P*<0.05) were used for two-dimensional hierarchical clustering using pearson correlation as similar metrics and complete linkage method for distance determination. Cluster 3.0 and Java TreeView 2.11 (Michael Eisen, http://rana.lbl.gov/EisenSoftware.htm) were applied for clustering and visualizing.

### Identification of Gadd45α direct interacting proteins

The Gadd45α direct interacting proteins were retrieved from Human Protein Reference Database (HPRD) (http://hprd.org/). The graphical output was generated using Cytoscape 2.6.0 (http://www.cytoscape.org/download.html).

### Animal and the establishment of STZ-induced diabetic rat model

Twelve male Wistar rats (weight 220–260 g) used in this study were obtained from the Animal Center of the Second Affiliated Hospital of Harbin Medical University (Harbin, Heilongjiang Province, China) and housed at controlled temperature of 23±1°C and humidity of 55±5%. Animals were maintained on 12-h dark-light artificial cycle (lights on at 07:00 A.M.) with food and water available *ad libium*. After fasting for 12 h, rats were intraperitoneally (ip) injected with 40 mg/kg of STZ in a 0.1 mol/l citrate buffer solution (pH 4.2) each day for 2 days, or the equal volume of citrate buffer for the rats in control group. Seventy-two hours after the final injection of STZ, the fasting blood glucose (FBG) level was determined from the tail vein of each rat using a Grace glucometer (Grace Medical, Inc., USA). The 6 STZ-induced rats with maintained FBG ≥16.7 mmol/l were used as established DM models. Four weeks after the induction of DM, all rats were killed for tissue harvesting.

### Quantitive real-time PCR (qRT-PCR)

After successfully construct the diabetic rat model, total RNA samples were isolated with Trizol (Invitrogen, Carlsbad, CA) from neonatal rat cardiac myocytes, left ventricle or nucleus ambiguous (NA) (−600 to +600 µm relative to obex) [14]. Then complementary DNA synthesis was performed using High-Capacity cDNA reverse transcription kits (Applied biosystems, Cat.# 4375222) according to the manufacturer's instructions with respective primers (rno-miR-499 RT primer: GTCGTATCCAGTGCGTGTCGTGGAGTCGGCAATTGCACTGGATACGACAAACAT; U6 RT primer: CGCTTCACGAATTTGCGTGTCAT, and Random primer). To quantify the target genes, qRT-PCR was performed with SYBR Green PCR Master Mix Kit (Applied biosystems, Cat.# 4309155) at 95°C for 10 min and 40 cycles at 95°C for 15 s, 60°C for 30 s and 72°C for 30 s on the 7500 FAST Real-Time PCR System (Applied Biosystems). U6 and β-actin were served as internal controls. The primers used were: Gadd45α (rat, NM_024127) sense: 5′-CCATAACTGTCGGCGTGT-3′ and antisense: 5′-CGCAGGATGTTGATGTCGT-3′; β-actin (rat, NM_031144.3) sense: 5′- ACTATCGGCAATGAGCG-3′ and antisense: 5′- GAGCCAGGGCAGTAATCT-3′; rno-miR-499 sense: 5′-GGGGTTAAGACTTGCAGTG-3′ and antisense: 5′-CAGTGCGTGTCGTGGAGT-3′; U6 sense: 5′- GCTTCGGCAGCACATATACTAAAAT-3′ and antisense: 5′- CGCTTCACGAATTTGCGTGTCAT-3′. The expression levels were compared with the value of Gadd45α and miR-499 normalized to the amounts of respective endogeneous controls (β-actin and U6). The expression levels were indicated with 2^−ΔΔCt^.

### Western Blot analysis

The protein expressions in neonatal rat cardiac myocytes, left ventricle or NA of rats were analyzed by Western blot analysis. The proteins under study were: Gadd45α (22 KDa) and β-actin (42 KDa). The protein content was determined with Sunrise-basic TECAN (Austria) using bovine serum albumin as the standard. Protein samples were fractionated by SDS-PAGE (10%–20% polyacrylamide gels) and transferred to PVDF membrane (Bio-Rad, Hercules, CA). Samples were incubated with primary antibodies specific for each protein (Santa Cruz, CA, USA) diluted at 1∶3000 in PBS buffer for 1 h at room temperature and then incubated with HRP-conjugated secondary antibodies diluted at 1∶5000 in the blocking buffer. Western blot bands were quantified using Odyssey v3.0 software (LI-COR Bioscience, Lincoln, NE, USA) by measuring the band intensity (area×OD) in each group and normalizing to β-actin and the final results were expressed as relative level by normalizing the data to the control values.

### MiRNA microarray data analysis and target prediction

The miRNA microarray experiment was performed by Kangcheng Bio-tech Inc. (Shanghai, China). Briefly, total RNA was extracted from left ventricle of diabetic and Ctl rats using Trizol reagent with quantity and purity of the RNA by UV absorbance and denaturing agarose gel electrophoresis. The samples (5 µg) were labeled using the miRCURY™ Hy3™ Array labeling kit (Exiqon, Cat# 208032), and hybridized in Hybridization Chamber II (Corning, Ambion, TX, USA, Cat# 40080) using miRCURY™ Array microarray kit (Exipon, Cat# 209002V8.1). After hybridization, the scanning was processed by a Genepix 4000B scanner (Molecular Devices, Downingtown, PA, USA). Data was analyzed by Genepix Pro 6.0 (Molecular Devices). To evaluate the significance of microarray results, random distribution was performed in MATLAB (R2008a) (http://download.cnet.com/Matlab/3000-2053_4-534283.html). After 100,000 times random sampling, *P*<0.05 was considered as significant difference to determine the differentially-expressed miRNAs. The miRNA expression levels from diabetic rats were compared with normal rats and the relative fold changes were calculated to present the up- or down-regulation of miRNAs. The miR-target pairs were predicted by MicroCosm Targets (Version 5, http://www.ebi.ac.uk/enright-srv/microcosm/htdocs/targets/v5/), Targetscan (Release 6.0, http://www.targetscan.org/), and PicTar (http://pictar.mdc-berlin.de/).

### Synthesis of various oligonucleotides

Rat miR-499 (GenBank acc. NR_032141) mimics (sense: 5′-UUAAGACUUGCAGUGAUGUUUGU-3′, antisense: 5′-AAACAUCACUGCAAGUCUUAAAU-3′), and rat miR-499 inhibitors (antisense oligonucleotides of mature miR-499, AMO-499: 5′-+A+C+A+A+ACATCACTGCAAGT+C+T+T+A+A-3′) were synthesized by Integrated DNA Technologies, Inc. (IDT, Coralville, IA, USA). The 5 deoxynucleotides at both ends of AMO-499 were locked (2′-O-methyl modifications at every base and a 4′-C-containing amino linker). In addition, a scrambled RNA was used as negative control (NC, sense: 5′-UUCUCCGAACGUGUCACGUTT-3′ and antisense: 5′- ACGUGACACGUUCGGAGAATT-3′).

### Cell culture and transfection

Neonatal rat cardiac myocytes were washed with serum-free medium and incubated in 6-well plates with 2 ml fresh fetal bovine serum (FBS). And then, cells (2×10^5^/well) were transfected with 50 nM miR-499, AMO-499 or negative control (NC) with X-treme GENE siRNA transfection reagent (Roche, Cat.# 04476093001) according to the manufacturer's instructions. Following transfection (48 h), cells were used for RNA or protein assay.

### Dual-luciferase Reporter Assay

Human embryonic kidney (HEK293) cells were cultured in DMEM supplemented with 10% FBS at 37°C with 5% CO_2_. The 3′UTR of Gadd45α holding miR-499 binding sites were cloned downstream of the luciferase reporter in pMIR-REPORT™ luciferase miRNA expression reporter vector (Ambion, Inc.). For luciferase assay, HEK293 cells were first starved in serum-free medium for 12 h, then transfected with 100 ng the chimeric plasmid (firefly luciferase vector), 20 ng PRL-TK (TK-driven Renilla luciferase expression vector) and 50 nM miR-499, AMO-499 or NC using X-treme GENE siRNA transfection reagent. Forty-eight hours after transfection, luciferase activities were measured with a Dual Luciferase Reporter Assay System (Promega, Cat.# E1960) on a luminometer (GloMax™ 20/20, Promega, USA).

### Statistical analysis

In biological experiments, data between two groups were expressed as mean ± SEM and compared using Student's *t*-test. *P*<0.05 was considered statistically significant.

## Results

### Co-differentially-expressed genes in DCM and DDRG

**Figure 1:**
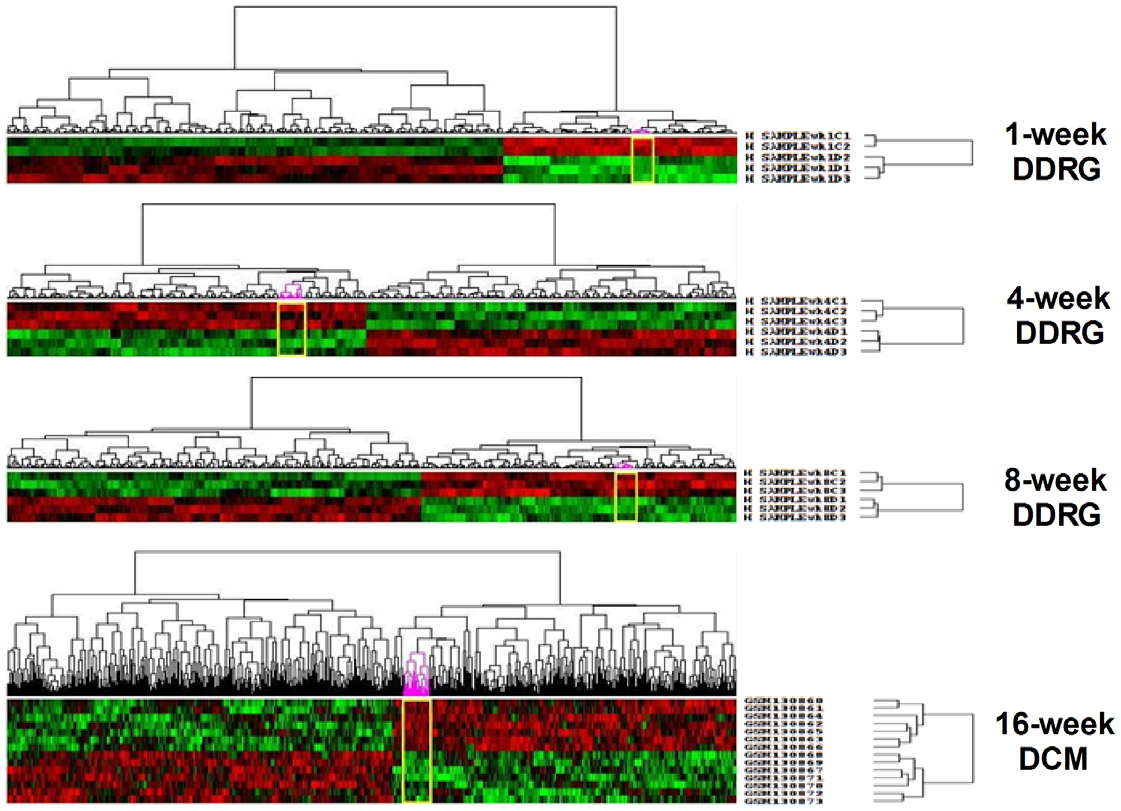
Co-differentially-expressed genes in 1, 4, and 8-week diabetic dorsal root ganglia (DDRG) and 16-week diabetic cardiomyopathy (DCM). Heat map represents data from the datasets of DDRG and DCM. Genes were identified by performing *t* tests on the large original dataset and comparing each diabetic group with the relative control group. Features with significantly different expression (*P*<0.05) were included. Heat map is organized with individual samples arranged along the Y-axis, and the relative ratios of expression were indicated by color. Color intensity is scaled as highest expression corresponding to bright red and the lowest expression corresponding to bright green. Heat map indicated that these genes were able to accurately classify different diabetic samples and relative controls, suggesting the representative roles of these genes in diabetes.

**Figure 2:**
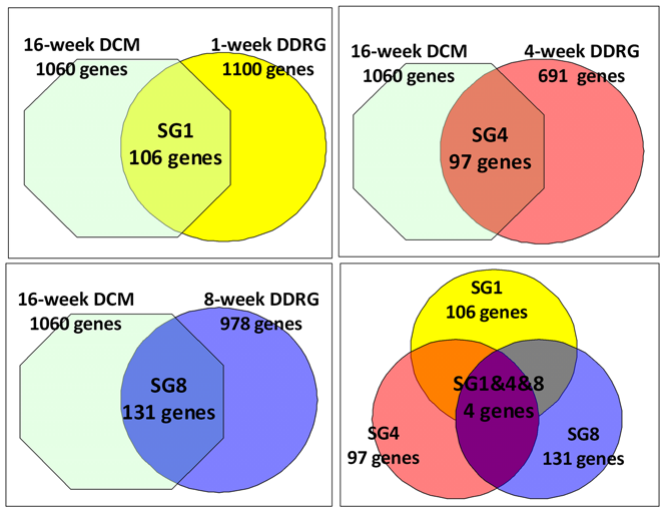
Identification of co-differentially-expressed genes between diabetic dorsal root ganglia (DDRG) and diabetic cardiomyopathy (DCM). Intersection analysis of differentially-expressed genes was performed to get shared genes 1 (SG1) between 16-week DCM and 1-week DDRG (A); shared genes 4 (SG4) between 16-week DCM and 4-week DDRG (B); shared genes 8 (SG8) between 16-week DCM and 8-week DDRG (C); and shared genes 1, 4 and 8 (SG1&4&8) among SG1, SG4 and SG8 (D).

By significance analysis of microarray, co-differentially-expressed genes of 1, 4 and 8-week DDRG as well as 16-week DCM were identified. As shown in Table 1, based on effective detected probes, the number of up- and down-regulated genes differed among these groups, which suggested the disparity of gene expression pattern in various tissues and time courses of DM. The heat maps after clustering algorithms for each pair of comparison were presented in Fig. 1, which well distinguished differentially-expressed genes in diabetic samples from relative controls. To further investigate the associations between differentially-expressed genes in DDRG and DCM, intersection analysis was carried out. As displayed in Fig. 2, we found 106 co-differentially-expressed genes in DCM and 1-week DDRG, 97 in DCM and 4-week DDRG, and 131 in DCM and 8-week DDRG. Notably, only the following 4 genes were identified in all the three previous intersection analysis: Gadd45α (growth arrest and DNA-damage-inducible, alpha), Ada (adenosine deaminase), Txnip (thioredoxin interacting protein), and Scn7a (sodium channel, voltage-gated, type VII, alpha). Considering the established effects of both Ada [15], [16] and Txnip [17] in DM and the largely undetermined biological function of Scn7a, we decided to focus on the role of Gadd45α in the crosstalk between DCM and diabetic baroreflex dysfunction.

**Table 1:** Differentially-expressed genes in 1, 4, and 8-week diabetic dorsal root ganglia (DDRG), and 16-week diabetic cardiomyopathy (DCM).

		Genes		
Group	Probes	Up-regulated	Down-regulated	Total
1-week DDRG	1750	748	352	1100
4-week DDRG	2765	350	341	691
8-week DDRG	2800	555	423	978
16-week DCM	1423	561	499	1060

### The direct interacting proteins of Gadd45α involved in DM

**Figure 3:**
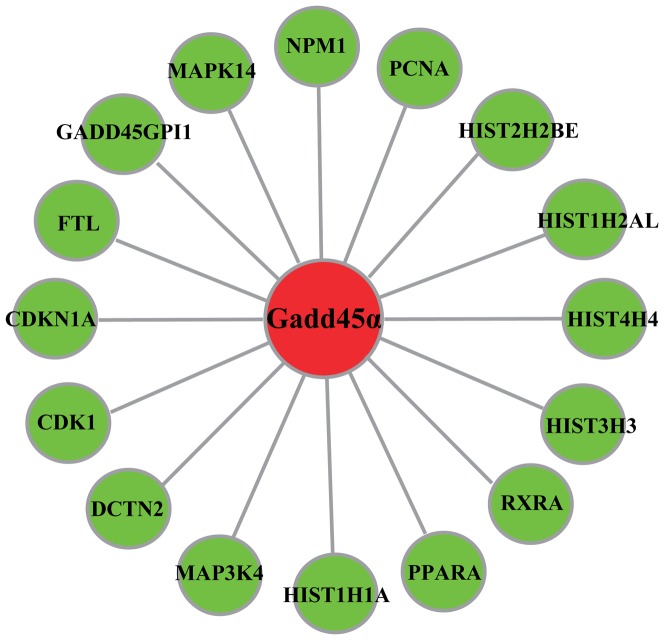
The direct interacting proteins of Gadd45α.

Although Gadd45α has been reported to participate in various physiological and pathological process [18], its role in DM has been seldom studied. In consideration with the complexity of DM, we herein presume that Gadd45α might be involved in a gene-interacting network and function with other DM-associated gene. As illustrated in Fig. 3, 16 direct interacting proteins of Gadd45α were identified by HPRD, and 11 of these direct interacting proteins have documented roles in DM (Table 2).

**Table 2:** The involvement of Gadd45α direct interacting proteins in diabetes mellitus.

Gene Name	Annotation	Reference
PPARA	peroxisome proliferator-activated receptor alpha	[28], [29]
PCNA	proliferating cell nuclear antigen	[44]
MAPK14	MAPK14	[45]
CDKN1A	Cyclin-dependent kinase inhibitor 1A	[31]–[34]
RXRA	Retinoid X receptor, alpha	[46]
CDK1	CDC2	[47]
Histone gene family (HIST1H1A/HIST1H2AL/HIST2H2BE/HIST3H3/HIST4H4)	Histone family (Histone 1, H1a/H2A histone family member 1/Histone 2, H2BE/Histon 3, H3/Histone 4, H4)	[25], [26]
MAP3K4	MAP3K4	Negative
NPM1	Nucleophosmin 1	Negative
DCTN2	Dynamitin	Negative
FTL	Ferritin light chain	Negative
GADD45GIP1	PRG6	Negative

### Altered Gadd45α expression in heart and NA from 4-week STZ-induced diabetic rats

**Figure 4:**
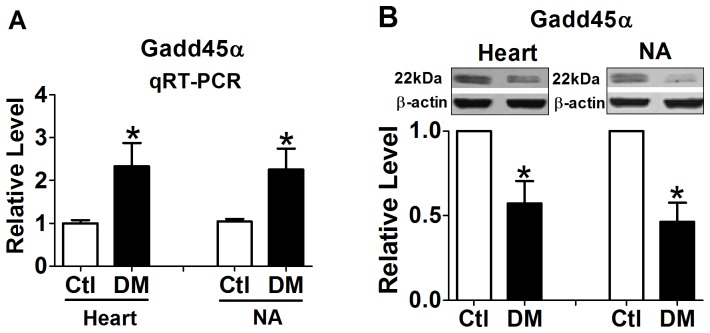
The Gadd45α mRNA (A) and protein expression (B) in diabetic heart and nucleus ambiguous (NA). *P<0.05 *vs* Ctl. Values are means of 6 independent experiments, with standard errors represented by vertical bars.

As NA is the exactly predominant component of baroreflex circuitry, we then validated the above bioinformatics results by analyzing Gadd45α expressions in heart and NA from 4-week STZ-induced diabetic rats. As shown in Fig. 4A, compared with Ctl samples, the mRNA expressions of Gadd45α were increased by 2.33±0.67 folds (*P*<0.05) and 2.26±0.60 folds (*P*<0.05) in diabetic heart and NA, respectively. However, the protein levels of Gadd45α were 0.57±0.16 fold (*P*<0.05) and 0.46±0.14 fold (*P*<0.05) in diabetic heart and NA compared with Ctl samples (Fig. 4B), which indicated the potential post-transcriptional mechanism might underlie the converse alternations in mRNA and protein levels.

### MiR-499 and Gadd45α, a co-differentially-expressed miR-target pair in heart and NA

**Figure 5:**
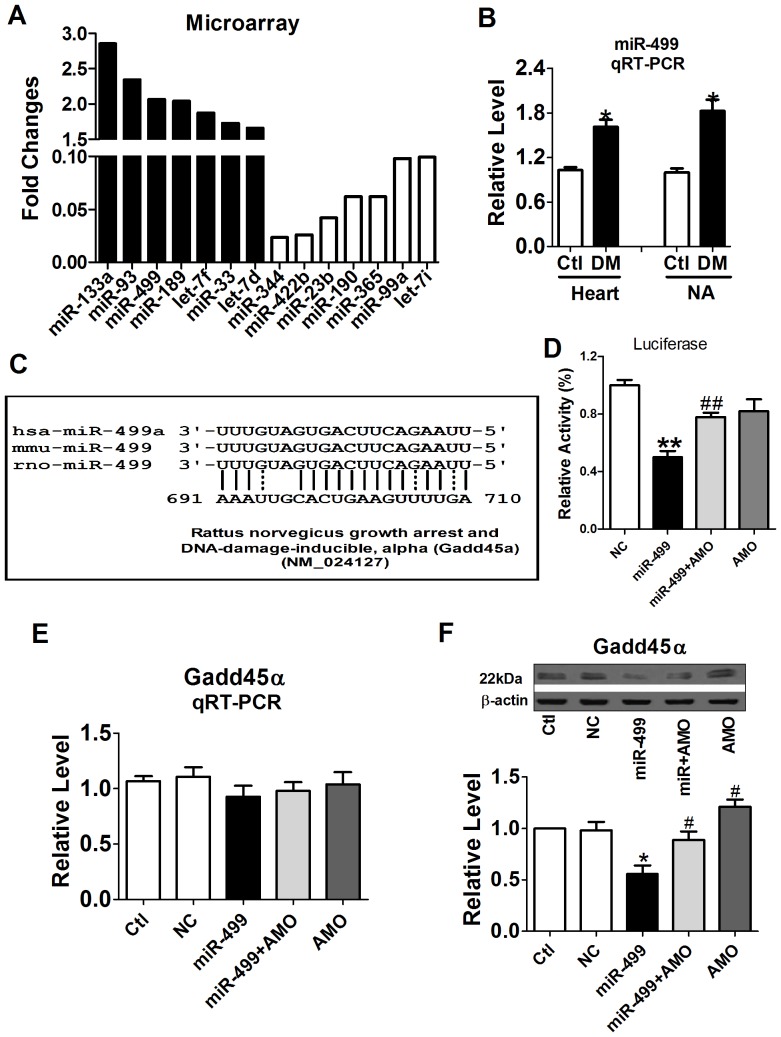
Increased expression of miR-499 in diabetic cardiomyopathy (DCM) and the effect of miR-499 on the expression of Gadd45α. (A) MiRNAs with significantly different expression (*P*<0.05) in DCM detecting by miR microarray (n = 3). (B) The expression of miR-499 was increased in diabetic heart and nucleus ambiguous (NA). *P<0.05 *vs* Ctl. (C) Complementarity between miR-499 and Gadd45α. (D) Suppression of miR-499 on the translation of Gadd45α by luciferase assay. The mRNA (E) and protein (F) expression of Gadd45α in miR-499 treated neonatal cardiac myocytes. *P<0.05 *vs* Negative control (NC). **P<0.01 *vs* NC; # *P*<0.05 *vs* miR-499; ##*P*<0.01 *vs* miR-499. Values are means of 6 independent experiments, with standard errors represented by vertical bars.

As miRNAs are well-known post-transcriptional factors, we speculated whether certain DM-induced differentially-expressed miRNAs underlie the altered Gadd45α expression in diabetic heart and NA. Left ventricles from diabetic and control rats were collected for miRNAs microarray analysis. Compared with Ctl samples, 7 up-regulated and 7 down-regulated miRNAs with significant changes (*P*<0.05) were presented in Fig. 5A. To identify the miRNAs with regulating effect on Gadd45α from these differentially-expressed miRNAs, 20 miRNAs being predicted to regulate Gadd45α by MicroCosm Targets, Targetscan, and Pictar were presented in Table 3. Furthermore, miRNAs microarray results indicated miR-499 was the single differentially-expressed miRNA among these regulating miRNAs of Gadd45α (Fig. 5A). Consistent with miRNAs microarray results, the subsequent qRT-PCR detection showed that DM led to miR-499 expression increased by 1.61±0.12 folds (*P*<0.05) and 1.83±0.18 folds (*P*<0.05) in diabetic heart and NA, respectively (Fig. 5B).

**Table 3:** Computationally predicted miRNAs targeting Gadd45α.

Predicted miRNA	MicroCosm Targets	TargetScan	PicTar
miR-101a*	Yes		
miR-130a	Yes		Yes
miR-130b	Yes		Yes
miR-134	Yes		
miR-148a			Yes
miR-148b-3p	Yes	Yes	Yes
miR-152	Yes	Yes	Yes
miR-219-2-3p	Yes		
miR-223	Yes		
miR-29b-1*	Yes		
miR-301a	Yes		Yes
miR-301b	Yes		Yes
miR-327	Yes		
miR-374	Yes		Yes
miR-376b-3p	Yes		
miR-499	Yes		
miR-7a*	Yes		
miR-871	Yes		
miR-881	Yes		
miR-883	Yes		

### MiR-499 negatively regulating Gadd45α

As miR-499 and Gadd45α displayed complementarity (Fig. 5C), luciferase analysis was performed to directly verify the regulating effect of miR-499 on Gadd45α. As shown in Fig. 5D, miR-499 transfection resulted in a notable decrease in luciferase activity of the chimeric luciferase vectors of Gadd45α (*P*<0.05, *vs* NC), which was significantly alleviated by co-transfected with AMO-499 (*P*<0.05, *vs* miR-499). To further investigate the biological effect of miR-499 on the Gadd45α expression, neonatal rat cardiac myocytes were used and transfected with miR-499, AMO-499 or NC. As demonstrated in Fig. 5E, transfection of miR-499 or AMO-499 showed no significant effect on the Gadd45α expression at mRNA level (*P*>0.05 *vs* NC). However, miR-499 significantly suppressed the protein expression of Gadd45α by 56% (*P*<0.05, *vs* NC), which could be partially reversed by co-transfection of AMO-499 (*P*<0.05, *vs* miR-499) (Fig. 5F). These results implied that miR-499 might repress Gadd45α expression by inhibiting transcription.

## Discussion

In the present study, by the combination of bioinformatics and biological experiments, we found that 11 proteins among 16 direct interacting proteins of Gadd45α are highly associated with DM. In addition, Gadd45α and miR-499 were co-differentially expressed in diabetic heart and NA, and Gadd45α is negatively regulated by miR-499. These findings suggest that the decreased Gadd45α protein level result from elevated miR-499 expression might potentially contribute to SCD in DM by their congenerous effects on diabetic heart and baroreceptor reflex.

DCM and baroreflex dysfunction were reported to be closely associated with SCD in DM [3], [4], and NA is an established predominant component of autonomic nervous system playing a vital role in heart rate control [19]. Therefore, NA and left ventricle of STZ-induced diabetic rats were studied for biological experiments to investigate tissue-tissue communication between DCM and diabetic baroreflex dysfunction in the present study. Although expression data of diabetic heart was acquired from GEO, we failed to get any expression data based on diabetic NA. Considering the similarity in organ development, a data profile deriving from STZ-induced diabetic neural system - DDRG was used to screen differentially-expressed genes. Co-differentially-expressed genes between DDRG and DCM were initially identified, and the hierarchical clustering of significantly differentially-expressed genes indicated an excellent distinction between disease and control groups (Fig. 1), which suggested the reliability of the criteria for our initial screening. Although there are hundreds of differentially-expressed genes in diabetic heart and DDRG at different time points in the course of the disease, four co-differentially-expressed genes (Gadd45α, Ada, Scn7a, and Txnip) were ultimately identified by intersection analysis between DDRG and DCM in all marked time points.

Consistent with our results, lines of reports have revealed the involvement of Txnip [17] and Ada [15], [16] in DM. Furthermore, Ada has been proposed as a potential biomarker of type 2 DM and diabetic nephropathy [20]. These studies provide sound evidence for the reliability of the co-differentially-expressed genes found in the present study. Although the biological function of Scn7a that encodes Na_x_ (a sodium channel of voltage-gated sodium channel family) [21] remains fragmental, our results prompt the potential role of Scn7a in DM-induced cardiovascular disease. Since studies on Txnip or Ada in DM are commonplace and no direct interacting proteins of Scn7a documented in HRPD led to difficulties in revealing its role in pathologies, we have not further investigated these genes.

Compared with the 3 genes discussed above, Gadd45α has been widely studied as a common expressed protein involved in cell growth, DNA repair and apoptosis [18]. The growth arrest- and DNA damage-induced (GADD) 45 protein family, comprising GADD34, GADD45α, GADD45β, GADD45γ, and GADD153, are widely expressed in mammalian cells and regulate coordinately. Amongst this family, GADD45α is predominantly nuclear localized, and responses to various cellular stresses and stimuli, including DNA damage, cellular senescence, apoptosis, oxidants, ionizing radiation, hypoxia, growth-factor deficiency, and ultraviolet light [22]. In addition, GADD45α is regulated by multiple transcriptional and post-transcriptional factors [22] and associated with lines of pathologies [23]. Nevertheless, little work has been done to demonstrate the role of Gadd45α in DM.

To further investigate the potential role of GADD45α in DCM and diabetic baroreflex dysfunction, we resorted to HPRD and identified its first-order interacting proteins in the present study (Fig. 3). As indicated in Table 2, histone-related and another 6 direct interacting proteins of GADD45α have been documented to be involved in DM, with examples as following: 5 members of histone gene family (Histone 1, H1a/H2A histone family member 1/Histone 2, H2BE/Histon 3, H3/Histone 4, H4) with potential participation in “histone code” hypothesis are pointed out to be associated with DM [24], [25], [26]; peroxisome proliferator-activated receptor alpha (PPARA) is dominantly located in the liver, kidney, and heart, and positively regulates the expression of genes implicated in fatty acid metabolism [27], the genetic variation at its locus is crucial to the incidence of diabetic cardiovascular complications [28], and its promoter variant was reported to decrease mortality after acute coronary ischemia in diabetic patients [29]; CDKN1A (cyclin-dependent kinase inhibitor 1A, p21, Cip1) encoding a protein widely binding to and repressing cyclin-cyclin-dependent kinase complexes is involved in carcinogenesis, senescence, and age-related diseases [30], and its de novomethylation induced by oxidative damage could function in the P53-dependent activation of cardiac cell death therefore contributes to the development of DCM [31], meanwhile it is also involved in diabetic nephropathy [32], β-cell glucose toxicity [33], and DM-associated vascular diseases [34].

These associations between the direct interacting proteins of GADD45α and DM established a regulating network of GADD45α, and provided circumstantial evidence that GADD45α might exert important functions by affecting its multiple direct interacting proteins in DCM and diabetic baroreflex dysfunction. Consequently, we further investigated the underlying mechanism of altered GADD45α expression under diabetic state. According to biological experimental results, we found the expression of Gadd45α was increased at mRNA level and decreased at protein level in both diabetic heart and NA. As miRNAs are short noncoding RNA molecules functioning by inhibiting translation or promoting mRNA degradation [7], we speculated the disparate mRNA and protein expressions of Gadd45α might be resulted from certain altered microRNAs expression. We then performed microRNAs microarray and found 14 differentially-expressed microRNAs in diabetic heart. Among these micoRNAs only miR-499 is computationally predicted to target Gadd45α.

MiR-499 has been established to reflect myocardial damage [35], [36], blunt the cardiac stress response [37], facilitate ventricular specification of human embryonic stem cells (hESCs) [38], be associated with cardiac differentiation [39], and regulate differentiation and proliferation in human-derived cardiomyocyte progenitor cells [40]. In addition, the expression of miR-499 was increased at circulating level after acute myocardial infarction [41], [42]. In the present study, increased miR-499 expression was verified by both miRNAs microarray analysis and qRT-PCR in diabetic samples. Interestingly, inconsistent with our observation of increased miR-499 in left ventricle and NA from 4-week STZ-induced diabetic rats, it is reported miR-499 expression was repressed in the retinas of 3-month STZ-induced diabetic rats [43]. The reversed alternations in miR-499 expression might be largely attributing to the different time courses (4 weeks *vs* 3 months) of DM.

In conclusion, increased miR-499 level accompanied with reduced Gadd45α protein expression was observed in the present study, which suggests that miR-499 :: Gadd45α might participate in the process of diabetic heart disease in STZ-induced diabetic rats. However, either miR-499 :: Gadd45α plays a key role or just acts as a cofactor without any critical influence in DCM and DM-induced baroreflex dysfunction needs to be further investigated separately in the future. Nonetheless, the differentially-expressed miR-target pair, miR-499 :: Gadd45α, was identified in DCM and DM-induced baroreflex dysfunction by the combination of bioinformatics and biological experiments. Furthermore, the numerous diabetes-associated direct interacting proteins of GADD45α indicated the importance of GADD45α in DM, and provide circumstantial evidence that GADD45α potentially acts as a linker between DCM and baroreflex dysfunction. Therefore, these findings establish a molecular foundation for further study on SCD in DM, and provide a new light on the predictive strategies for SCD in DM.
